# Efficiency and Flexibility of Fingerprint Scheme Using Partial Encryption and Discrete Wavelet Transform to Verify User in Cloud Computing

**DOI:** 10.1155/2014/351696

**Published:** 2014-09-24

**Authors:** Ali A. Yassin

**Affiliations:** Computer Science Department, Education College for Pure Science, Basrah University, Basrah 61004, Iraq

## Abstract

Now, the security of digital images is considered more and more essential and fingerprint plays the main role in the world of image. Furthermore, fingerprint recognition is a scheme of biometric verification that applies pattern recognition techniques depending on image of fingerprint individually. In the cloud environment, an adversary has the ability to intercept information and must be secured from eavesdroppers. Unluckily, encryption and decryption functions are slow and they are often hard. Fingerprint techniques required extra hardware and software; it is masqueraded by artificial gummy fingers (spoof attacks). Additionally, when a large number of users are being verified at the same time, the mechanism will become slow. In this paper, we employed each of the partial encryptions of user's fingerprint and discrete wavelet transform to obtain a new scheme of fingerprint verification. Moreover, our proposed scheme can overcome those problems; it does not require cost, reduces the computational supplies for huge volumes of fingerprint images, and resists well-known attacks. In addition, experimental results illustrate that our proposed scheme has a good performance of user's fingerprint verification.

## 1. Introduction

Recently, we are witnessing an increasing interest in cloud computing. Many Internet vendors including Amazon, Google, and Microsoft have introduced various cloud solutions to provide computing resources, programming environments, and software as services in a pay-as-you-go manner. For example, Amazon introduces Amazon Elastic Compute Cloud (EC2) which provides computing cycle as a service and Google introduces Google App Engine (GAE) to provide programming environments as a service [1, 2].

Cloud has raised many concerns related to security, data leakage, and sharing of resources. As the heart of the security concern, authentication plays an essential role in the current cloud computing systems [3].

Our work focuses on two security fields. The first one is fingerprint verification while the second field represents partial encryption of digital image based on discrete wavelet transformation (DWT).

### 1.1. Fingerprint Verification

Biometrics-based individual authentication schemes that apply physiological biometric such as eye recognition, fingerprint, or behavioral actions such as speech and handwriting traits are becoming progressively widespread compared to conventional systems that are depending on e-tokens (e.g., RSA-token) or knowledge such as password [4]. Conventional authentication systems cannot distinguish between an adversary that illegally gains the access privileges of an honest user and the sincere user himself. Moreover, biometric authentication schemes can be more appropriate for the users since there is no password to fail to recall or token to be stolen/lost and a single biometric trait such as fingerprint can be used to access many accounts without needing to remember their passwords. Additionally, fingerprints are insistent with age and cannot be easily discriminated. Consequently, fingerprint is one of the most studied and matured fields of biometric authentication. Fingerprint identification is considered as one of the most renowned and disseminated biometrics. It includes a set of ridge lines, which primary flow parallel, producing a pattern. The points of ridge lines terminate or bifurcation are named minutiae [5–7] whereas permitting to Galton, each ridge is categorized by several minute individualities called minutiae, which may split and almost directly reunite, enclosing a small spherical or elliptical space or sometimes the autonomous beginning or termination of ridges. The ridges are represented by dark while valleys are connected by bright. Ridges and valleys often run in parallel; sometimes they bifurcate or they terminate some other times. Finally, a good quality fingerprint must contain 25–80 numbers of minutiae [6] according to the sensor resolution and finger location on the sensor. Briefly, fingerprint technique considers a complex pattern recognition issue; designing algorithms that have the ability to extract significant features and match them in a strong approach is fairly hard, especially in low quality fingerprint images. There is a common fallacy that self-propelled fingerprint recognition is a completely solved issue since it became in the first level of pattern recognition applications almost fifty years ago. On the other side, fingerprint technique is still a challenging and a vital pattern recognition issue.

As a result, the fingerprint verification system consists of two phases: enrollment and verification. In enrollment phase, the user registers his fingerprint as a template to the server who extracts features from user's fingerprint. At the verification phase, the fingerprint data of a user is compared with the template stored in the server and then it detects the validity of a user. We notice traditional fingerprint system required extra hardware and software for each user's logging system. In this paper, we proposed a new scheme for verifiying user without required software for main stages: input fingerprint, preprocessing image, features extraction, classification, and verification. Additionally, our proposed scheme does not need to use the extra hardware for reading user's fingerprint for each user's logging system.

### 1.2. Image Encryption

Cryptography is considered as one of the technical field that means to support security to data being communicated on information and communications structures. It is especially beneficial in the states of monetary and user's data, irrespective of the actuality that the user's data is being sent over a medium or is saved on a storage device [8]. It supports a powerful means of validating the authenticity of data and recognizing the culprit, if the authority and integrity of the data are desecrated. Because of the improvement of electronic business, cryptographic methods are hardly critical to the improvement and usage of defiance information technologies and communications networks. Dissimilar to text messages, digital image data have their special characteristics, such as bulk capacity, high correlation among pixels, and high redundancy, not to indicate that they commonly are enormous which together make conventional encryption schemes difficult to use and slow to process [9]. The major aim of the partial encryption is to abbreviate the computational workload and performance and to obtain a good security level.

Image encryption algorithms can be classified into full encryption and partial encryption depending on the sum of the encrypted data. Regrettably, the performance of encryption and decryption functions is a major concern in real-time image transmission. Time can be classified into two ranks, the first rank of encryption time and the second rank of time transferring images. Encryption and decryption functions are not fast enough to deal with the vast amount of transmitted data. An important criterion connecting to the scheme is to reduce the image encryption size and keep quality of the image. Partial encryption method is an appropriate scheme to encrypt only the lowest segment of data to reduce the computational requirements of huge amounts of multimedia data. It is necessary to reduce the performance of images encryption in distributed network by minimizing the sum of data to encrypt, attaining a logical security, and minimizing the computation [10, 11].

### 1.3. Our Contributions

In this paper, we propose a new scheme for user's fingerprint authentication, solving the issues in the ordinary setting. In particular, the motivations of our work are fivefold.

First, we analyze the disadvantages of traditional user's fingerprint authentication. To make our analysis solid, we support our analysis on this generic construction.

Second, we merge three factors (fingerprint, partial image encryption, and DWT) for proposing a new scheme based on user's fingerprint verification.

Third, our proposed scheme does not require extra hardware (fingerprint scanner, e-token) and software (input fingerprint, preprocessing, feature extraction, classification, matching).

Fourth, our proposed scheme has a good balance between security and performance of fingerprint verification comparing with previous schemes.

Fifth, our proposed scheme contains important merits as follows: (1) it provides mutual verification between user and service provider; (2) it accomplishes user anonymity; (3) the service provider and the user can achieve verified session's keys; (4) it has several advantages such as low cost, simple integration with available infrastructure, and essay to deploy and manage in practical system.

### 1.4. Organization

The rest of this paper is organized as follows. The necessary primitives and requirements of our scheme exist in Section 2. An overview of related work is displayed in Section 3. Our proposed scheme is presented in Section 4. We detail the security analysis and implementation results in Section 5, and Section 6 concludes the paper.

## 2. Design Issues

### 2.1. Problem Definitions

**Figure 1:**
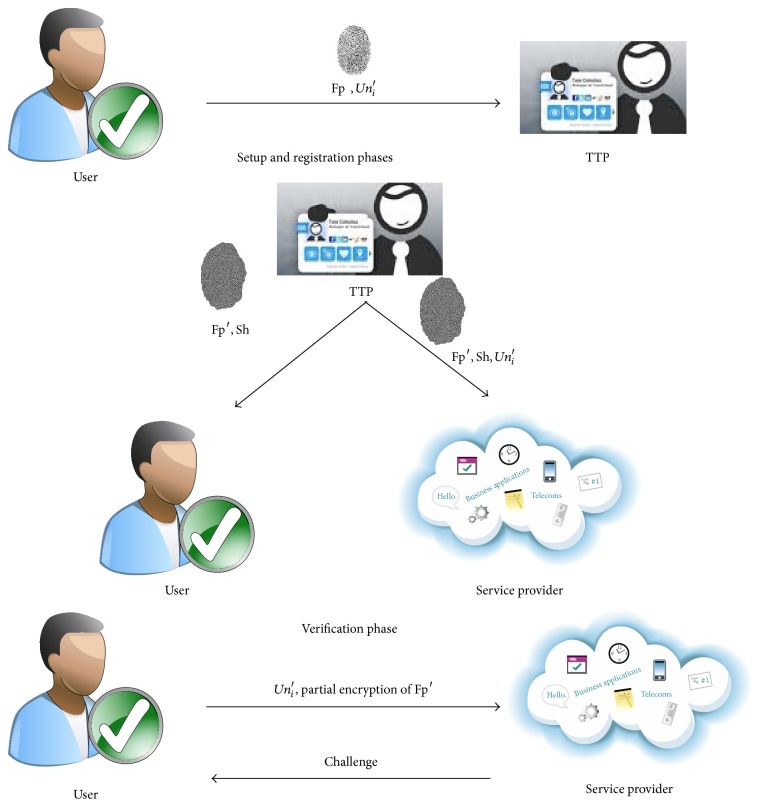
View the main phases of our proposed Scheme.

Figure 1 explains the main idea of our proposed scheme, which consists of three components:* trusted third party* (TTP),* service provider* (SP), and users (*U*_*i*_). TTP is responsible for gaining main keys to each user valid () and service provider () to complete verification phase. Generally, the proposed scheme can be divided into three phases: setup, registration, and verification. In our proposed scheme, *U*_*i*_ will register their username (*Un*_*i*_′ = *h*(*Un*_*i*_)) and fingerprint (Fp) to TTP in the setup phase. After that, TTP computes discrete wavelet transform on user's fingerprint Fp′ = DWT(Fp) and generates shared key (Sh). Then, it sends important information (Fp′, Sh, *Un*_*i*_′), (Fp′, Sh) to the service provider and user, respectively. However, user's important information and service provider's important information are shared in (Fp′, Sh). After that, *U*_*i*_ encrypts and saves his important information in any extra device that prefers it. In verification phase, the valid user sends their username anonymity and encrypts their fingerprint Fp′ based on (DWT, Sh) to SP. Then, SP verifies user's securing information to check the validity of user depended on important information that has received at setup phase from TTP. Then, SP sends back challenge value to the user for ensuring validity of SP. As a result, SP allows the user to access resources which he wishes to use.

### 2.2. Discrete Wavelet Transform

**Figure 2:**
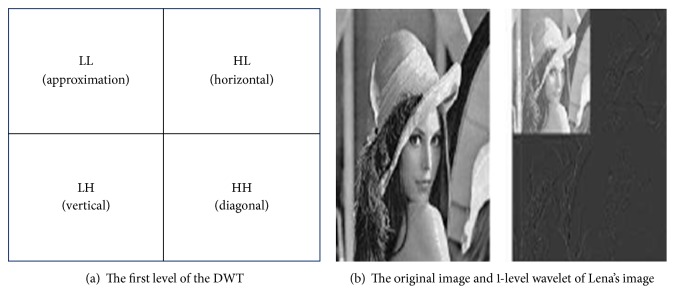
Apply DWT on Lena's image.

The discrete wavelet transform is used to analyze the two-dimension image where it has the ability to divide the image into four main areas. The distributed data depends on the degree of the important data. The data area is called the low-low (LL) subband. Occasionally, it is named as an approximation area. The rest of the regions are known as the detail. The present subbands are as follows: high-low (HL), low-high (LH), and the high-high (HH). In brief, they are indicated as DWT [11, 12]. The wavelet analysis is permitted in using the long periods of time where the ambition is to get more accurate information from low frequency and high frequency from the shorter zones [9–12]. The important data reside in the low frequency because it gives you the opportunity to get a signal of an identity. When you go to the high frequency content, it is characterized by high accuracy. It can be observed by the wavelet analysis for Lena's image (see Figure 2(a)).  Figure 2(b) displays the first level of the DWT [13, 14]. The tactic followed by the separation of the different characteristics of the signal relies on the method of gathering the energy of a few elements. This is called mechanism subband coding. This class of DWT refers to the place of analysis via the approximation area at level *j* in four zones. Specifically, level *j* + 1 consists of approximation and the details. The details are distributed in three zones: horizontal, vertical, and diagonal [15].

### 2.3. Histogram

**Figure 3:**
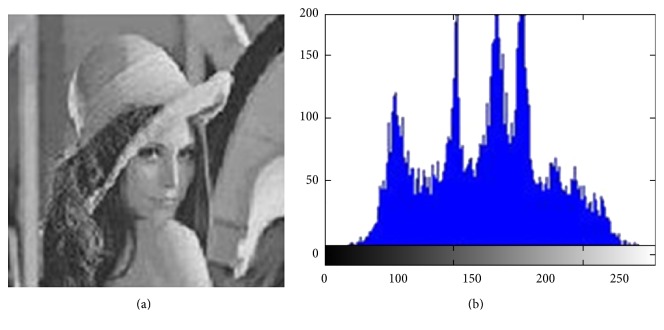
Histogram of Lena's image.

A histogram image is the mathematical standard of the digital image. Furthermore, it helps to understand the distribution of the graphic image. The histogram image means the process of distribution density of brightness and the contrast in the gray-level image [9, 10]. This method is applied in our approach as measure; anyone can note enhanced ratio during this scale.  Figure 3 refers to the image of the histogram method.

### 2.4. Correlation

Correlation (Crr) is a measure of sameness between the image before and after processing. The exemplary outcomes are near the one which can be identified by the following equation [16–19]:

(1)  Crr  =(∑r=1N∑c=1M(In(r,c)−In−)(I0(r,c)−In0−))  ×([∑r=1N∑c=1M(In(r,c)−In−)2]kkkkkkkk[∑r=1N∑c=1M(In(r,c)−In−)2]×[∑r=1N∑c=1M(In(r,c)−In−)2])−1,

where *I*_*n*_(*r*, *c*) is the digital value of the pixel in the (*r*, *c*) of image before processing and *I*_*n*_ is referring to the image before processing:

(2) $$\begin{array}{c} \overset{-}{I_{n}}=\frac{1}{M\times N}\overset{N}{\underset{r=1}{\sum }}\;\overset{M}{\underset{c=1}{\sum }}\left(\overset{-}{I_{n}}\left(r,c\right)\right). \end{array}$$

*I*_0_(*r*, *c*) is the digital value of the pixel in the (*r*, *c*) of image before processing:

(3) $$\begin{array}{c} \overset{-}{I_{0}}=\frac{1}{M\times N}\overset{N}{\underset{r=1}{\sum }}\;\overset{M}{\underset{c=1}{\sum }}\left(\overset{-}{I_{0}}\left(r,c\right)\right), \end{array}$$

where  *M* is height of the image, *N* is width of the image, and *r* and *c* are row and column numbers.

The average of these correlations is used to generate the Crr of the recreated color image in RGB system. The color correlation equation is as follows:

(4) $$\begin{array}{c} {\text{Crr}}_{\text{RGB}}=\frac{{\text{Crr}}_{\text{Red}}+{\text{Crr}}_{\text{Green}}+{\text{Crr}}_{\text{Blue}}}{3}. \end{array}$$

## 3. Related Work

### 3.1. Image Encryption

Information privacy is an essential feature of digital image encryption. The privacy of the encrypted data with stability in time and cost effectiveness of the encryption method is the main challenge that is faced in image encryption topic. There are many related works that referred to this challenge [9–17] distributing via the frequency, spatial, and the hybrid field methods in full encryption schemes.

Panigrahy et al. [18] propose image encryption scheme using the Hill cipher. They are constructing self-invertible matrix for Hill cipher scheme. Then, they used this key matrix to encrypt gray level as well as color images. Their scheme works fine for all types of digital images excluding the images with background of the same gray scale or same color. Additionally, their scheme required time processing for full image encryption.

Nien et al. [19] applied a hybrid encryption technique for the true image depending on the multichaotic model. They combined the* pixel-chaotic-shuffle* (PCS) and* bit-chaotic-rearrangement* (BCR) schemes. The first one (PCS), a fast encryption scheme which can vary the locations of each pixel, is practical to fully disregard the original image outlines applying four third-order chaos like Henon, Lorenz, Chua, and Rȍssler chaos maps. The second one (BCR) employs chaos scheme to make chaotic codes reorganization in pixels practical. The merging of the PCR and the BCR will cause us to increase the key space of digital images and suffers from resisting the extensive attack when correlation coefficient is refers to 0.0031 [17].

Seyedzade et al. [20] presented an algorithm for image encryption depending on SHA-512 hash function. Their algorithm divides into two major fields. The first one does preprocessing procedure to shuffle one half of digital image while the second one applies hash function to create a random number mask. Then, the mask is XORed with the other section of the image that is going to be encrypted.

Abuhaiba and Hassan [21] propose a new image encryption scheme which employs amount and phase manipulation applying* differential evolution* (DE) method. Their scheme has passed key out of space analysis, algebraic analysis, and key sensitivity analysis to exhibit the security of the new image encryption scheme. Also, this scheme requires high performance to complete encryption/decryption function.

Munir [22] presents a technique that uses the Arnold cat map transformation on low frequency subband of the DCT transformed to encrypt image. Their essential idea for choosing the low frequency subband of the DCT transformed image is certified to the truth that the* human visual system* (HVS) is more imagined to important information (such as object, shape) at lower frequencies compared with higher frequency information. Their scheme is represented to be strong against noise, though to some amount since the decrypted image views some attendance of noise.

In this paper, we use partial image encryption based on discrete wavelet transform detection as the main factor to verify legal user in cloud computing environment. TTP sends important information (discrete wavelet transform of user's fingerprint, shared key, and user's anonymity) to valid user and cloud service provider. The shared key derives from discrete wavelet transform of user's fingerprint and uses then it uses it to encrypts/decrypts image. Additionally, our proposed scheme embeds salt key with shared key to generate one time key for each user's login. Also, our work encrypts user's fingerprint once time for each verification phase. In the performance our presented scheme has been evidenced to achieve sturdy security with low cost and high performance compared with previous schemes.

### 3.2. Fingerprint Verification

**Figure 4:**
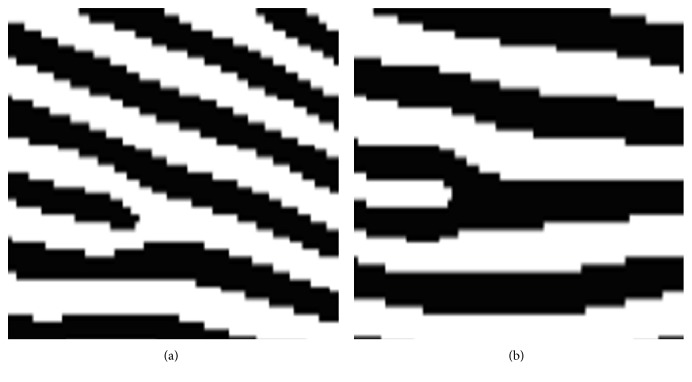
Minutiae points. (a) Ridge ending. (b) Bifurcation.

The wide majority of modern automated fingerprint authentication schemes are minutiae (level 2 features) using biometric systems [6, 7]. The two most distinguished kinds of minutiae are ridge termination and ridge bifurcation [23]. The minutiae-based systems are generally implemented well with high-accuracy fingerprint images and an adequate fingerprint surface area. These conditions, however, may not be attainable at any time. In several cases, only a small part of the test fingerprint can be comparative with the reference fingerprint. Additionally, comparing fingerprints gained via small-area sensors is hard due to the potentiality of having too little interference between different gains of the same finger [24]. Furthermore, the image of fingerprint is a unique pattern that consists of ridge and valley points on the surface of a finger. The ridge is well defined as a single curved section, and a valley is the region between two adjacent ridges. Minutiae points (see Figure 4) represent the local ridge cavities, which are classified into two classes: bifurcations and ridge endings. A good quality fingerprint image has minutiae from 40 to 100. Continuously, these minutiae points are employed to detect the uniqueness of a user's fingerprint. Ultimately, the minutiae consider the first part processes a raw fingerprint image and extracts fingerprint features from that image.

On the other side, there are several schemes based on pattern recognition of fingerprint image as follows.

Arora and Singla [25] presented a scheme that depended on fingerprint verification which has been advanced using* Laboratory Virtual Instrument Engineering Workbench* (LabVIEW). The proposed verification scheme consists of two phases: enrollment and authentication. The proposed scheme uses a learning stage during the enrollment phase, which does not exist in conventional image-depending systems. In the learning stage a pseudorandom subsampling is achieved in which pixels are analyzed by testing their surrounding neighborhood for regularity and each pixel is categorized according to how large the regularity of its surrounding neighborhood is (e.g., 3 × 3, 5 × 5, etc.). This stage will reduce the amount of computations in the matching phase. In the verification phase, user sends his username and password as a first factor. After that, the user submits his fingerprint image to check user's validity. This scheme suffered from malicious attacks.

Chen et al. [26] presented rebuilding the fingerprint's orientation field from minutiae and employ it in the matching phase to enhance the system's performance. Cao et al. [27] have presented two novel characteristics to deal with nonlinear deformation in fingerprints. These characteristics are the finger appointment direction and the ridge compatibility. Choi et al. [28] presented incorporating ridge characteristics such as ridge count, ridge curvature direction, ridge length, and ridge type together with minutiae to increase performance of the matching phase.

In this paper, we propose a secure and efficient fingerprint verification scheme for the cloud computing using partial encryption of fingerprint based on discrete wavelet transform. In this paper, we proposed a new scheme for verifying user without required software for main stages: input fingerprint, preprocessing image, feature extraction, classification, and verification. Additionally, our proposed scheme does not need to use the extra hardware for reading user's fingerprint for each user's logging system.

## 4. Our Proposed Scheme

**Figure 5:**
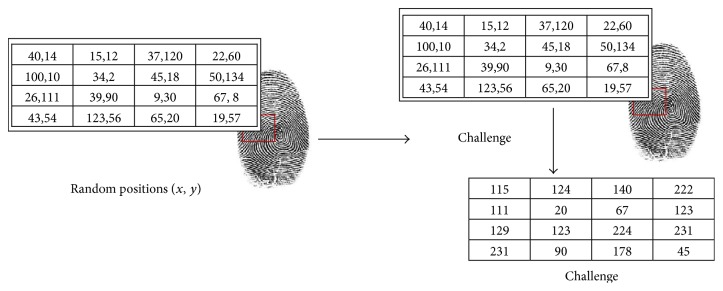
The mechanisim of computing challenge.

The common notations in Table 1 will be used throughout this paper. Our proposed scheme consists of three phases—setup, registration, and verification. In the setup phase, the major elements (TTP, SP, and *U*_*i*_) also use a cryptographic hash function *h*(·) and a symmetric key encryption/decryption Enc(·)/Dec(·), and TTP sets up *n* = *p*∗*q*, where both *p* and *q* are two large primes. The user (*U*_*i*_) sends his username (*Un*_*i*_ = *h*(username)) and fingerprint Fp_*i*_ to the trusted third party (TTP) through a secure channel. After that, TTP computes Fp_*i*_′ = DWT(Fp_*i*_) where DWT is used to analyze the two-dimension image where it has the ability to divide the image into four main areas. The distributed data depends on the degree of the important data. The data area is called the low-low (LL) subband. Occasionally, it is named as an approximation area and represents Fp_*i*_′. After that, TTP saves (*Un*_*i*_, Fp_*i*_′) and then provides public parameters keys PK = (*Un*_*i*_, Fp_*i*_′, Sh ∈ *Z*_n_^*^) and secret keys SK = (Fp_*i*_′, Sh ∈ *Z*_n_^*^) to service provider and each user, respectively, in the secure channel. Lastly, *U*_*i*_ encrypts his secret keys (SK′ = Enc_Sh_(SK)) by using his shared key and then saves his encrypted file at his preferred storage such as USB, Samsung mobile. After registration phase, the user can use his secret key to log in to the system. The verification session is qualified as follows (see Figure 5).

**Figure 6:**
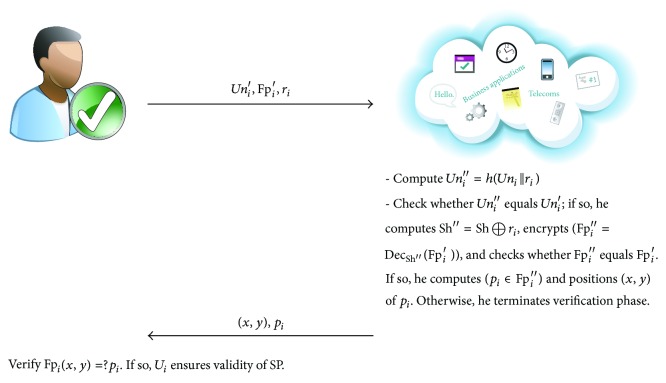
The verification phase of our proposed scheme.

(1) *U*_*i*_ → *SP* : *Un*_*i*_′, Fp_*i*_′, *r*_*i*_. *U*_*i*_ performs the following steps.
  (i) Decrypt his secret keys file based on his shared key SK = Dec_Sh_(SK′).
  (ii) Generate random number *r*_*i*_ ∈ *Z*_n_^*^ and compute one time anonymous username *Un*_*i*_′ = *h*(*Un*_*i*_∣∣*r*_*i*_), once shared key Sh′ = Sh ⊕ *r*_*i*_, and (Fp_*i*_′ = Enc_Sh′_(Fp_*i*_)).
  (iii) Send *Un*_*i*_′, Fp_*i*_′, *r*_*i*_ to SP as a first factor.
(2) *SP* → *U*_*i*_ : (*x*, *y*), *p*_*i*_.  SP checks the validity of user's information as follows.
  (i) Compute *Un*_*i*_′′ = *h*(*Un*_*i*_∣∣*r*_*i*_) and check whether *Un*_*i*_′′ equals *Un*_*i*_′. If so, he accepts the user's information request and performs the next step. Otherwise, SP terminates verification phase.
  (ii) SP computes Sh′′ = Sh ⊕ *r*_*i*_, encrypts (Fp_*i*_′′ = Dec_Sh′′_(Fp_*i*_′)), and checks whether Fp_*i*_′′ equals Fp_*i*_′. If so, he accepts the user's information request and performs the next step. Otherwise, SP terminates verification phase.
  (iii) SP generates a random number from user's fingerprint (*p*_*i*_ ∈ Fp_*i*_′′) and positions (*x*, *y*) of *p*_*i*_ as a challenge to valid user for ensuring validity of SP (see Figure 6).
(3) User verifies Fp_*i*_(*x*, *y*) = ?*p*_*i*_. If it holds, then *U*_*i*_ ensures validity of SP.

## 5. Experimental Results

### 5.1. Security Analysis

In this section, we offer the security analysis of our proposed scheme. We will show that our scheme is secure against well-known attacks such as MITM attack, replay attack, and insider attack. Moreover, our proposed scheme enjoys several merits, such as containing user anonymity, mutual verification, and session key agreement.

**Theorem 1 .**

The proposed scheme can supply mutual verification.

**Proof**

The mutual verification feature requires that an attacker cannot impersonate a legal user to the service provider, and vice versa. Only the valid user has the secret information (*Un*_*i*_′, Fp_*i*_′, *r*_*i*_, Sh) to send these parameters to the service provider. SP compares (*Un*_*i*_′, Fp_*i*_′) with (*Un*_*i*_′′, Fp_*i*_′′), respectively. If so, a user is verified. To complete mutual verification, the user verifies Fp_*i*_(*x*, *y*) with *p*_*i*_. If it holds, then *U*_*i*_ ensures validity of SP. Therefore, our proposed scheme achieves mutual verification between the two entities (see Figure 6).

**Theorem 2 .**

The proposed scheme can supply user's anonymity.

**Proof**

If an adversary attempts to eavesdrop on the user's login request, he cannot find the user's anonymity from crypto hash function *Un*_*i*_′ = *h*(*Un*_*i*_∣∣*r*_*i*_) since it is embedded in a random number *r*_*i*_ with username, which are not identified to an adversary. Additionally, *r*_*i*_ generates once for each user's login request. Hence, it is difficult for an adversary to disclose the user's password. Clearly, our proposed scheme can support user's anonymity.

**Theorem 3 .**

The proposed scheme can provide security of the stored data.

**Proof**

In the proposed scheme, only user's secret data (SK) is stored on user's storage such as USB, Samsung phone. The data of SK does not help an adversary to use it without the user's shared key (Sh). If the legal user loses his preferred storage, it is difficult for any adversary to access or update the credential information because he cannot obtain the user's secret key (SK). Therefore, the user's secret data protects from retrieving by an adversary.

**Theorem 4 .**

The proposed scheme can support known key security and session key agreement.

**Proof**

In our proposed scheme, when the user sends his parameters to log in to the the service provider, he generates secret key Sh′ = Sh ⊕ *r*_*i*_ to encrypt discrete wavelet transform of his fingerprint which is represented by Fp_*i*_′ = Enc_Sh′_(Fp_*i*_). Even if an adversary can access the previous session key, he is still unable to get fresh values of Sh′ which generates once for each user's login session. So an adversary cannot compute the new session key. Continuously, the service provider can obtain the same new key Sh′′ = Sh ⊕ *r*_*i*_ based on secret parameters (Sh, *p*_*i*_) to compute Fp_*i*_′′ = Dec_Sh′′_(Fp_*i*_′). Finally, SP checks the validity of user's fingerprint based on Sh′′. Additionally, our proposed scheme depends on user's fingerprint to obtain secret keys (Sh′, Sh′′) that are derived by using ((*x*, *y*), *p*_*i*_). So an adversary cannot get these secret parameters.

**Theorem 5 .**

The proposed scheme can forward secrecy.

**Proof**

Our proposed scheme preserves the user's fingerprint even when the secret key Sh′ is disclosed or leaked. If the secret key is revealed by the adversary, the verification of the system is not impressed, and he cannot use this key in the next login phase. At the same time, it is extremely hard that an adversary can derive the secret key which consists of random number and shared key Sh. An adversary still cannot obtain the secret key Sh which is used to encrypt user's fingerprint Fp_*i*_′ = Enc_Sh′_(Fp_*i*_). Hence, our work maintains the forward secrecy.

**Theorem 6 .**

The scheme can prevent a replay attack.

**Proof**

An adversary performs a replay attack by eavesdropping the login message which is sent by a legal user to the service provider. Then, an adversary reuses this message to impersonate the user when logging into the system in a next session. In our proposed scheme, each new user's login request should be identical with SP's keys (*Un*_*i*_′, Fp_*i*_′, *r*_*i*_, Sh′). Therefore, an adversary cannot pass any replayed message to the SP's verification. Moreover, our work can resist this attack without synchronization clocks. So, our scheme depends on random *r*_*i*_ instead of timestamp. As a result, an adversary fails to apply this type of attack.

**Theorem 7 .**

The scheme can resist a reflection attack.

**Proof**

This type of attack happens when a valid user submits his login message to the service provider; the adversary tries to catch user's message and send it (or an updated version of the message) back to the same user. In our proposed scheme, the adversary fails to cheat the service provider since he cannot restore the pair ((*Un*_*i*_′′, Sh′), Fp′) sent from the user via verification phase. The service provider and user have one-time secret keys (Sh′, Sh′′) to compute (Fp_*i*_′, Fp_*i*_′′), respectively. Thus, our proposed scheme prevents the reflection attack.

**Theorem 8 .**

The proposed scheme can resist the MITM attack.

**Proof**

This type of attack is intended when an adversary has the ability to intercept the messages between users and the server. Then, he uses this message when the user signs out from the server. In our proposed scheme, the factors are securely encrypted and sent to the service provider. Generation of the random value *r*_*i*_ is through the creation of sensitive data (Sh′, Fp′) by *U*_*i*_ as a login request to SP. This sensitive data becomes useless when *U*_*i*_ signs off from the server. Therefore, an adversary spotting communication between *U*_*i*_ and SP can learn *r*_*i*_ which is used only once; he is unable to compute Sh′, Fp′. However, when *U*_*i*_ signs out of the service provider, an adversary cannot compute both factors to impersonate the genuine user. As a result, the proposed scheme can resist MITM attack.

**Theorem 9 .**

The proposed scheme can provide revocation.

**Proof**

In case of lost or stolen information of user's preferred storage such as USB, *U*_*i*_ will present request to SP for its revocation by pushing his information (*Un*_*i*_′, Fp_*i*_′). SP verifies the user's secure information and ensures whether *Un*_*i*_′′ = *Un*_*i*_′ and *Pp*_*i*_′ = *Pp*_*i*_′′. If the result is valid, SP deletes registration of user's file (SK = (Fp_*i*_, Sh, *n*) from registration table. Lastly, the user can change his username and password by reperforming the registration phase. Additionally, an adversary cannot get any benefits from stealing user's extra storage because he cannot be tolerated to decrypt registration user's file which requires from an adversary to obtain user's fingerprint Fp_*i*_. Obviously, our proposed scheme can provide revocation.

### 5.2. Implementation and Results

**Figure 7:**
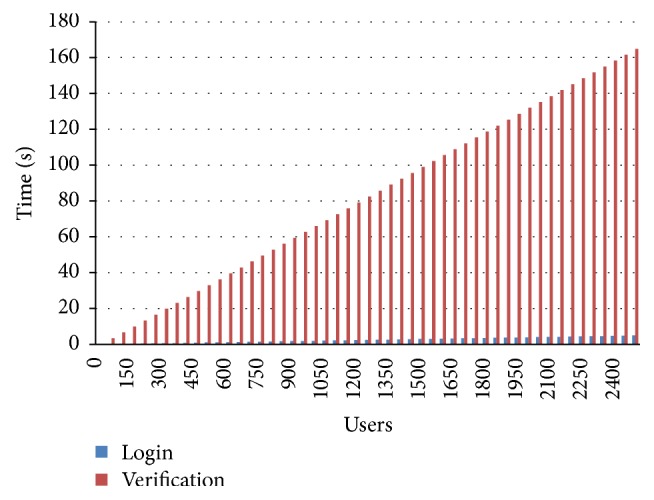
The performance of our proposed scheme.

In this section, we conduct several experiments for gauging the efficiency and the effectiveness of our work.  Figure 7 shows time processing of verification and login phases. However, the average time for the login and verification phase of our work is equal to 0.135601 seconds for each user who indicates the high speed of our solution. The estimation parameters are declared in Table 2. The time requirement of our scheme is brief in Table 3. We have registered during our experiments 2500 users and supposed that each user needs maximum 0.75 seconds for logging into the system. Our proposed scheme uses CASIA Fingerprint Image Database Version 5.0 (or CASIA-FingerprintV5 [29]) that consists of 20,000 user's fingerprint images of 500 topics. The users' fingerprint images of CASIA-FingerprintV5 were taken using URU4000 fingerprint sensor in one session. The volunteers of CASIA-FingerprintV5 include graduate students, employees, and waiters.

**Figure 8:**
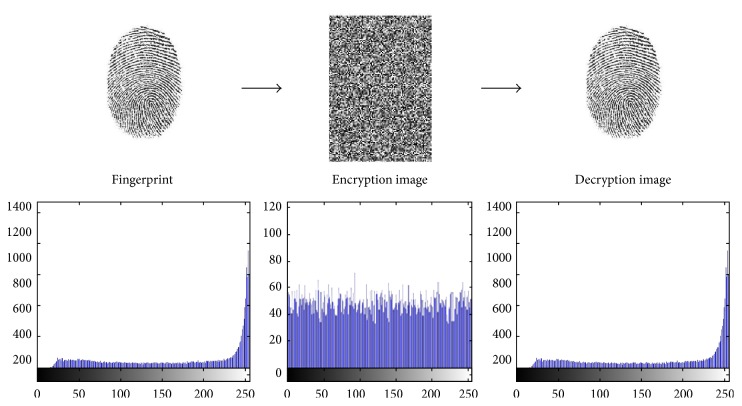
The main stages of encryption/decryption of User 1's fingerprint.

**Figure 9:**
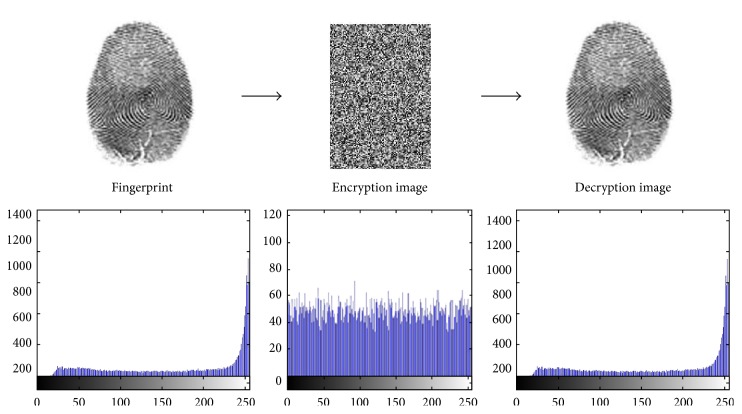
The main stages of encryption/decryption of User 2's fingerprint.

**Figure 10:**
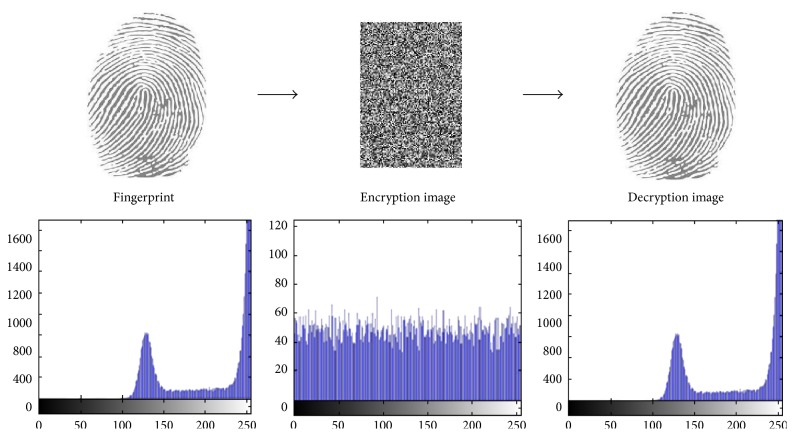
The main stages of encryption/decryption of User 3's fingerprint.

**Figure 11:**
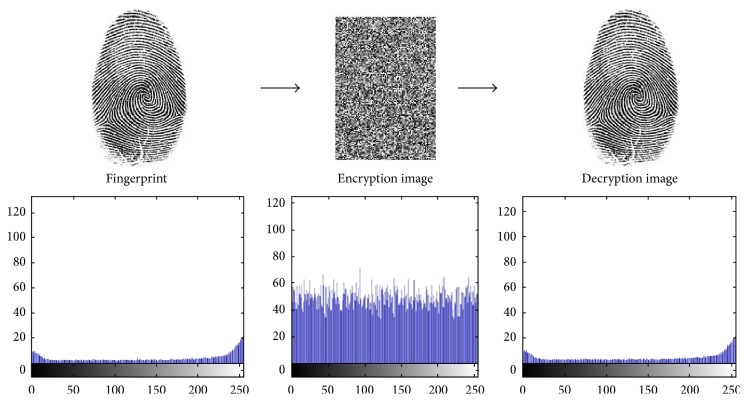
The main stages of encryption/decryption of User 4's fingerprint.

In partial image encryption of discrete wavelet transform of fingerprint, we demonstrate the experimental results of the first two users' fingerprints of different sizes with color image. To prove the sturdiness of our proposed scheme, we have implemented statistical analysis by computing the histograms, entropy, and the correlation of two adjacent pixels in the original image/encrypted image. The results of experimental and statistical analysis show that our proposed scheme supports an efficient and secure manner for real-time image encryption and transmission. Figures 8, 9, 10, and 11 explain histogram of original, encrypted, and decrypted images. In addition to that, Table 4 shows the results of correlation and entropy of original, encrypted, and decrypted images.

## 6. Conclusion

In this paper, we have proposed a new fingerprint verification scheme for cloud computing environment that includes one-time username anonymity and partial image encryption based on edge detection as a second factor. Our proposed scheme aims to support more functionality and to resist familiar attacks. These vital merits include the following: (1) the valid user can freely choose his passwords; (2) our proposed scheme supports mutual verification between service provider and legal user; (3) it achieves one-time username anonymity; (4) service provider and legal user can produce verified sessions keys; (5) our proposed scheme can provide revocation and security of the stored data. Moreover, our scheme can resist the stolen-verifier problem, reflection attacks, replay attacks, forgery attacks, and parallel session attacks. On the other side, partial encryption is considered one of the most promising clarifications to reduce the cost of data protection in communication network. In this paper, we proposed a good concept of pixel value manipulation using discrete wavelet transform for fingerprint partial encryption scheme. From the viewpoint of security, the experimental results explain that our proposed scheme achieves better security than the individual encryption schemes. The proposed scheme has a good performance, the highest entropy, and the lowest correlation compared to the present partial encryption schemes. Hence, better security has been supported via several theorems that are presented at Security Analysis Section.

## Figures and Tables

**Table 1:** Notations of our proposed scheme.

Symbol	Definition
*h*(·)	A cryptographic hash function
Enc(·)/Dec(·)	A symmetric key encryption/decryption function
*n*, *p*, *q*	Large primes numbers
*Un*_*i*_, *Un*_*i*_′, *Un*_*i*_′′	User anonymity
Fp_*i*_	User's fingerprint
Sh	Shared key
PK	Public parameters provided by TTP to SP at setup phase
SK	Secrete keys provided by TTP to *U*_*i*_ at setup phase
Fp_*i*_′, Fp_*i*_′′	Partial encryption of user's fingerprint
*r*_*i*_, SK′, *K*_*i*_	Other miscellaneous values which are used in the verification
*p*_*i*_	A random pixel selected from user's fingerprint
(*x*, *y*)	The positions (*x*, *y*) of pixel (*x*, *y*)
DWT	Discrete wavelet transform

**Table 2:** Estimation parameters.

Symbol	Definition
*T*_*h*_	Time processing of a hash function
*T*_Xor_	Time processing of Xor function
*T*_Opr_	Time processing of mathematical operations such as multiplication, addition, and subtraction
*T*_Enc_	Time processing of symmetric encryption operation
*T*_Dec_	Time processing of symmetric decryption operation
*T*_||_	Time processing of concatenation function
*T*_DWT_	Time processing of DWT function

**Table 3:** Performance of our proposed scheme.

Phase	TTP	User	Service provider
Setup and registration	T_DWT_ + T_Opr_	T_h_ + T_Enc_	—
Verification	—	2T_Opr_ + T_Enc_ + T_Dec_ + T_Xor_ + T_||_ + T_h_	T_Opr_ + T_Dec_ + T_Xor_ + T_h_ + T_||_

Total	T_DWT_ + T_Opr_	2T_Opr_ + 2T_Enc_ + T_Dec_ + T_Xor_ + T_||_ + 2T_h_	T_Opr_ + T_Dec_ + T_Xor_ + T_h_ + T_||_

**Table 4:** Correlation and entropy of encrypted/decrypted image.

Original image	Correlation	Entropy of encryption image	Entropy of decryption image
Fingerprint of User 1	0.1211	7.9895	1.6469
Fingerprint of User 2	0.0158	7.9857	1.6102
Fingerprint of User 3	0.265	7.9857	1.2656
Fingerprint of User 4	0.4102	7.9869	2.8941
